# Case-study: Patient with second depressive episode and catatonia

**DOI:** 10.1192/j.eurpsy.2026.11424

**Published:** 2026-07-31

**Authors:** I. Grechenliev, P. Terziivanova

**Affiliations:** 1 Second Psychiatric Clinic, University Hospital for Neurology and Psychiatry “Sveti Naum”; 2 Department of Psychiatry and Medical Psychology, Medical University - Sofia, Sofia, Bulgaria

## Abstract

**Introduction:**

Catatonia is a neuropsychiatric syndrome with a multifactorial etiology and varied clinical presentation. Timely recognition of patients as catatonic is all too frequently hindered by the initial diagnostic uncertainty, as catatonia commonly develops on the backdrop of general medical, neurological, or other psychiatric conditions. Additionally, the presence of a catatonic syndrome itself may often obscure underlying neurological or somatic disease processes. Accordingly, proper delineation of strictly catatonic phenomena from other causes of motor dysfunction is essential for adequate treatment of such patients. Depression is among the more common psychiatric conditions manifesting with catatonic episodes.

**Objectives:**

A 65 year-old female with Major depressive disorder was referred to the emergency service due to changes in his psychomotor activity observed by her relatives.

**Methods:**

We used medical history, clinical observation, and Bush-Francis Catatonia Rating Scale (BFCRS), 23-item version.

**Results:**

The case of a 65-year-old а female with catatonia will be presented to elucidate some of the more poignant diagnostic and treatment challenges. Of note are the anxious-depressive onset of the disease, the predominantly antidepressive treatment prior to the catatonic exacerbation, as well as the ambiguous neurological findings.

**Conclusions:**

The case presentation attempts to compare and contrast the condition of the patient with the myriad of closely-related motor abnormalities that can confound the clinician. Lastly, the condition will be reviewed in light of possible alternative explanations. Neurodegenerative, hormonal, pharmacogenic and other hypothetical causes will be examined in order to better illustrate the numerous differential diagnostic possibilities that regularly feature when discussing such a complex neuropsychiatric condition.

**Disclosure of Interest:**

None Declared

